# High-Coverage Profiling of Hydroxyl and Amino Compounds in Sauce-Flavor Baijiu Using Bromine Isotope Labeling and Ultra-High Performance Liquid Chromatography–High-Resolution Mass Spectrometry

**DOI:** 10.3390/metabo15070464

**Published:** 2025-07-09

**Authors:** Zixuan Wang, Youlan Sun, Tiantian Chen, Lili Jiang, Yuhao Shang, Xiaolong You, Feng Hu, Di Yu, Xinyu Liu, Bo Wan, Chunxiu Hu, Guowang Xu

**Affiliations:** 1 State Key Laboratory of Medical Proteomics, Dalian Institute of Chemical Physics, Chinese Academy of Sciences, Dalian 116023, China; wzx@dicp.ac.cn (Z.W.); chentiantian@dicp.ac.cn (T.C.); yudi_1808@dicp.ac.cn (D.Y.); liuxy2012@dicp.ac.cn (X.L.); xugw@dicp.ac.cn (G.X.); 2 University of Chinese Academy of Sciences, Beijing 100049, China; 3 Liaoning Province Key Laboratory of Metabolomics, Dalian 116023, China; 4 Key Laboratory of Quality and Safety of Jiangxiangxing Baijiu, State Administration for Market Regulation, GuiZhou XiJiu Co., Ltd., Zunyi 564622, China; sunyoulan022@163.com (Y.S.); jianglili202506@163.com (L.J.); yuhaoshang909@163.com (Y.S.); youxiaolong1122@163.com (X.Y.); hufeng2025xj@163.com (F.H.)

**Keywords:** sauce-flavor Baijiu, hydroxyl and amino compounds, chemical derivatization, bromine isotope labeling, UHPLC-HRMS, food metabolomics

## Abstract

**Background**: Hydroxyl and amino compounds play a significant role in defining the flavor and quality of sauce-flavor Baijiu, yet their comprehensive analysis remains challenging due to limitations in detection sensitivity. In this study, we developed a novel bromine isotope labeling approach combined with ultra-high performance liquid chromatography–high-resolution mass spectrometry (UHPLC-HRMS) to achieve high-coverage profiling of these compounds in sauce-flavor Baijiu. **Methods**: The method employs 5-bromonicotinoyl chloride (BrNC) for rapid (30 s) and mild (room temperature) labeling of hydroxyl and amino functional groups, utilizing bromine’s natural isotopic pattern (Δ*m*/*z* = 1.998 Da) for efficient screening. Annotation was performed hierarchically at five confidence levels by integrating retention time, accurate mass, and MS/MS spectra. **Results**: A total of 309 hydroxyl and amino compounds, including flavor substances (e.g., tyrosol and phenethyl alcohol) and bioactive compounds (e.g., 3-phenyllactic acid), were identified in sauce-flavor Baijiu. The method exhibited excellent analytical performance, with wide linearity (1–4 orders of magnitude), precision (RSD < 18.3%), and stability (RSD < 15% over 48 h). When applied to sauce-flavor Baijiu samples of different grades, distinct compositional patterns were observed: premium-grade products showed greater metabolite diversity and higher contents of bioactive compounds, whereas lower-grade samples exhibited elevated concentrations of acidic flavor compounds. **Conclusions**: These results demonstrate that the established method is efficient for the comprehensive analysis of hydroxyl and amino compounds in complex food matrices. The findings provide valuable insights for quality control and flavor modulation in sauce-flavor Baijiu production.

## 1. Introduction

Chinese Baijiu, one of the world’s six major distilled spirits, has earned global recognition for its complex flavor profiles and cultural significance. Among its various types, sauce-flavor Baijiu represents the most characteristic variety [1], produced through traditional solid-state fermentation of sorghum and wheat followed by distillation and aging [2]. During fermentation, microbial metabolism generates a vast array of hydroxyl and amino compounds, including alcohols, phenols, amines, and amides, which collectively shape its sensory attributes and bioactive properties [3]. For instance, higher alcohols contribute to a mellow taste [4]; phenolic derivatives impart smoky and floral aroma profiles [5]; while amides enhance nutty aromatic notes and amines suppress fruity and floral aromas [6]. Moreover, certain hydroxyl and amino metabolites exhibit physiological activities [5,7]. Comprehensive analysis of hydroxyl and amino compounds in Baijiu is thus critical for optimizing flavor quality, ensuring product consistency, and exploring bioactive functionalities.

Current analytical methods for Baijiu components predominantly rely on gas chromatography–mass spectrometry (GC-MS), which excels in volatile compound detection [8,9,10] but struggles with polar, non-volatile, or thermally labile hydroxyl/amino metabolites. Liquid chromatography–mass spectrometry (LC-MS) offers broader coverage but faces sensitivity limitations due to the poor ionization efficiency of some hydroxyl and amino compounds [10,11,12]. Chemical derivatization has emerged as a powerful strategy to enhance LC-MS performance by improving the chromatographic retention and ionization efficiency of these compounds. Numerous derivatization methods have been specifically developed for hydroxyl and amino compounds. For instance, Markus Bösl et al. designed a novel derivatization reagent, dimethyl-glycidyl-tolyl-ammonium (DGTA), for analyzing volatile alcohols in food products [13]. Similarly, Guo et al. established a dansyl chloride derivatization method for profiling amine and phenolic metabolites in human urine [14]. However, most of the existing derivatization protocols require elevated temperatures or prolonged reaction times, risking analyte degradation and compromising analytical throughput. Additionally, isotope labeling approaches for compound screening, while effective, necessitate costly heavy isotope reagents and extensive standard libraries, restricting their practical applications in complex matrices like Baijiu. Consequently, there is an urgent need to develop a derivatization reaction with milder and faster reaction conditions.

The identification of hydroxyl and amino compounds derivatized by chemical labeling reagents typically involves two main steps, preliminary screening and structural annotation. For compound screening, chemical isotope labeling (CIL) strategies have been widely employed [15,16,17]. In these approaches, light and heavy isotope-labeled tags are introduced into target and reference compounds, respectively, via derivatization reactions. The resulting isotope-labeled compound pairs produce mass spectral peaks with fixed mass differences at identical chromatographic retention times, enabling efficient screening of labeled compounds. Despite the fact that CIL significantly improves screening efficiency and reduces false positives during annotation, it requires the additional synthesis of heavy isotope-labeled derivatization reagents, thereby increasing labor and cost. Regarding the compound annotation, reference libraries based on derivatized standards are commonly used. For instance, the Li group constructed the MyCompoundID library, which comprises 315 dansylated compounds [18]. The Feng group developed multiple derivatization strategies targeting different functional groups and established the Chemical Labeling Metabolite Database (CLMD) [19]. However, the acquisition of standard compounds is labor-intensive and costly, and the annotation scope remains limited. Therefore, there is an urgent need for more efficient, convenient, and cost-effective strategies for the screening and annotation of labeled compounds.

In this study, we proposed a high-coverage analytical method for hydroxyl and amino compounds in sauce-flavor Baijiu using bromine isotope labeling coupled with ultra-high performance LC–high-resolution MS (UPLC-HRMS). A novel brominated derivatization reagent, 5-bromonicotinoyl chloride (BrNC), was designed to enable the efficient labeling of hydroxyl and amino compounds under ambient conditions without the need for heating or prolonged reaction times. The distinctive natural isotopic signature of bromine (1:1 ratio for [M]^+^/[M + 2]^+^) served as an intrinsic mass marker for automated compound screening. Subsequently, the defined compounds were annotated according to retention time (t_R_), accurate mass (MS1), and MS2 spectra. Using this approach, a total of 309 hydroxyl and amino compounds were successfully characterized in sauce-flavor Baijiu. The established method was further employed to investigate the distribution characteristics of these compounds in sauce-flavor Baijiu of different quality grades.

## 2. Materials and Methods

### 2.1. Chemicals and Reagents

Acetonitrile (ACN) (HPLC-grade), ethanol (HPLC-grade), and dichloromethane (DCM) (HPLC-grade) were purchased from Merck (Darmstadt, Germany). Ultra-pure water was prepared by Milli-Q system (Millipore, Bedford, MA, USA). Formic acid (HPLC-grade) and 4-dimethylaminopyridine (DMAP) were purchased from J&K (Beijing, China). 5-bromonicotinoyl chloride was purchased from Bidepharm (Shanghai, China). Dansyl chloride and benzoyl chloride were purchased from J&K (Beijing, China). All standards of hydroxyl and amino compounds were purchased from Sigma-Aldrich (St Louis, MO, USA).

### 2.2. Preparation of Stock Solutions and Calibrators

Stock solutions of individual hydroxyl and amino standards were prepared separately in ACN and ultra-pure water at a concentration of 2 mg/mL. A mixed standard working solution was prepared by combining equal volumes of these individual stock solutions, followed by serial dilution to obtain final concentrations of 10,000, 5000, 2500, 1250, 500, 250, 100, 50, 25, 12.5, 5, 2.5, 1, 0.5, 0.25, 0.125, 0.05, 0.025, 0.01, 0.005, 0.0025, and 0.00125 ng/mL. Benzyl alcohol-d7, Sorbitol-^13^C6, L-Phenylalanine-d5, and L-Leucine-^13^C were employed as internal standards (ISs) and were individually prepared in ACN at a concentration of 2 mg/mL.

### 2.3. Sampling and Sample Preparation

All Baijiu samples (SFB1, SFB2, SFB3, SFB4, SFB5) were obtained from local markets. Both Baijiu and blank samples (53% ethanol aqueous solution) were concentrated 25-fold using a vacuum rotary evaporator (100 rpm/min, 45 °C, and −0.1 MPa) and stored at 4 °C until analysis. Quality control (QC) samples were prepared by mixing equal volumes of all Baijiu samples. Detailed sample information is provided in Table S1 in the Supporting Information.

For sample preparation, 30 μL of concentrated Baijiu or blank sample was mixed with 5 μL of internal standard solution in a 1.5 mL Eppendorf tube. Subsequently, 300 μL of DCM and 150 μL of ultra-pure water were added to the mixture. The mixture was vortexed at 4 °C and 1500 rpm for 5 min and then subjected to ultrasonication in an ice-water bath for 5 min. The samples were then centrifuged at 15,000 rpm and 4 °C for 10 min. The aqueous and organic phases were separately transferred into two 1.5 mL EP tubes and freeze-dried.

For chemical derivatization, 100 μL of ACN, 10 μL of BrNC suspension (10 mg/mL in ACN), and 10 μL of DMAP solution (10 mg/mL in ACN) were sequentially added to the freeze-dried samples. The mixture was then vigorously vortexed for 30 s at room temperature. Subsequently, 20 μL of water was added to quench the excess derivatization reagent. The samples were then freeze-dried and reconstituted in 50 μL of ACN/water (1:1, *v*/*v*) for UPLC-HRMS analysis.

To compare the derivatization efficiency of BrNC, DNS, and BenCl, the following procedures were employed: (i) For DNS derivatization, sequentially add 50 μL of DNS acetonitrile solution (25 mg/mL) and 50 μL of NaHCO_3_/Na_2_CO_3_ buffer (pH 8.0) to the lyophilized standard mixture. Incubate the reaction at 40 °C for 60 min. (ii) For BenCl derivatization, sequentially add 50 μL of BenCl acetonitrile solution (2%, *v*/*v*) and 50 μL of NH_4_HCO_3_ buffer (100 mM) to the lyophilized standard mixture. Vortex the mixture at room temperature for 30 s. (iii) For BrNC derivatization, sequentially add 100 μL of ACN, 10 μL of BrNC suspension (10 mg/mL in ACN), and 10 μL of DMAP solution (10 mg/mL in ACN) to the lyophilized standard mixture. Vortex the mixture at room temperature for 30 s.

### 2.4. Establishment of Quantitative Structure–Retention Relationship (QSRR) Model

A total of 82 standard compounds were selected for the development and evaluation of the QSRR model. Following the recommendations from the QSRR_Automator software user guide [20], 62 standards were randomly chosen as the training set, while the remaining 20 were assigned to the test set. The retention times (t_R_) of all BrNC-derivatized standards were acquired using UPLC-HRMS. The SMILES representations of all compounds in the training set, along with their BrNC-derivatized t_R_, were imported into QSRR_Automator to construct the QSRR model, followed by internal cross-validation. The established QSRR model was then applied to predict the t_R_ of the BrNC-derivatized compounds in the test set, and the average absolute error (AAE) between the predicted and actual t_R_ values was calculated to assess the reliability of the model. The model was finally employed to predict the t_R_ of the BrNC-derivatized compounds in the database.

### 2.5. UPLC-HRMS Analysis

Chromatographic separation was conducted using a 1290 series UHPLC system (Agilent, Santa Clara, CA, USA) equipped with an ACQUITY BEH C8 column (2.1 mm × 100 mm, 1.7 μm; Waters, Milford, MA, USA). The column temperature was maintained at 50 °C, and the flow rate was set to 0.35 mL/min. The mobile phase consisted of 0.1% (*v*/*v*) formic acid in water (Phase A) and 0.1% (*v*/*v*) formic acid in ACN (Phase B). The gradient elution program was initiated with 5% B for 1 min, followed by a linear increase to 100% B over 23 min, which was then held for 4 min. Subsequently, the gradient was reduced to 5% B within 0.1 min and equilibrated for 1.9 min.

The untargeted MS data were acquired using a 6546 series quadrupole time-of-flight (Q-TOF) MS system (Agilent, Santa Clara, CA, USA) operating in positive ion mode. The spray voltage was set at 4.0 kV. The desolvation gas flow rate was maintained at 8 L/min, with a temperature of 320 °C, while the sheath gas flow rate was 11 L/min at 350 °C. Full-scan mass spectra were recorded over an *m*/*z* range of 100–1100 Da. Auto-MS/MS analysis was performed under the following conditions: collision energies of 10, 20, and 40 eV; a maximum of five precursors per cycle; a precursor threshold of 1000 counts; and an MS2 scan range of *m*/*z* 50–1000 Da.

### 2.6. Performance Evaluation of the Method

To evaluate the reliability of the established analytical method, a comprehensive assessment was conducted, including linearity range, limit of quantification (LOQ), limit of detection (LOD), intra-day and inter-day precision, repeatability, and stability. A standard mixture comprising 10 hydroxyl and amino compounds was utilized for this purpose. To assess linearity, a series of standard working solutions were prepared, as described in Section 2.2, with concentrations ranging from 0.00125 to 10,000 ng/mL. Specifically, 100 μL of each working solution was mixed with 10 μL of an IS solution at a concentration of 1 μg/mL. The resulting mixture was subsequently freeze-dried before undergoing BrNC-labeling. Linearity was determined by constructing a regression curve and plotting the IS-normalized peak areas of the labeled standard compounds against their respective concentrations. The LOD and LOQ were defined as the lowest injection concentrations at which the signal-to-noise ratios reached 3 and 10, respectively.

To assess intra-day and inter-day precision, blank samples were spiked with the standard mixture at three concentration levels (low, medium, and high). Intra-day precision was evaluated by analyzing six replicates of the prepared samples within a single day (*n* = 6), while inter-day precision was assessed by analyzing triplicate samples over three consecutive days (*n* = 3 × 3). Additionally, method repeatability was determined by processing six blank samples spiked with a 500 ng/mL standard solution in parallel (*n* = 6 × 3).

The stability of BrNC-labeled compounds over a 48 h period was also examined. Briefly, a blank sample spiked with a 100 ng/mL standard solution was processed and stored at 4 °C following derivatization. The peak areas of the labeled compounds were measured at 0, 8, 24, 32, and 48 h. The relative standard deviations (RSDs) of the IS-normalized peak areas were calculated to evaluate the stability of the derivatives over a two-day period.

### 2.7. Data Processing

Peak detection was carried out using Agilent MassHunter Qualitative Analysis 10.0 (Agilent, Santa Clara, CA, USA), while peak alignment and integration were conducted using Agilent MassHunter Profinder 10.0 (Agilent, Santa Clara, CA, USA). The peak list, including t_R_, peak area, average *m*/*z*, and MS2 spectra, was generated using MS-DIAL 4.24. The QSRR model was constructed using QSRR_Automator. The in-silico MS2 spectra were generated by CFM-ID [21]. An in-house Python script was employed to identify bromine isotope mass spectrum peak pairs. Another in-house Python script was utilized to search multiple databases, including an in-house Baijiu database, the Yeast Metabolome Database (YMDB) (Yeast Metabolome Database), and the Collective Molecular Activities of Useful Plants (CMAUP) (CMAUP—Collective Molecular Activities of Useful Plants), for compound annotation. The Python script was written and executed using Spyder 3.12.

### 2.8. Strategy for Screening and Annotating Hydroxyl and Amino Compounds

The complete annotation workflow for hydroxyl and amino compounds in Baijiu is illustrated in Figure S1. Following the acquisition of untargeted UPLC-HRMS data, BrNC-derivatized compounds were initially screened based on the characteristic isotope peak pairs introduced by bromine atoms. Specifically, an in-house Python script was employed to identify features with a fixed mass difference (Δ*m*/*z* = 1.998 Da ± 10 ppm) and a predefined intensity ratio (0.8–1.2), which were subsequently included in a candidate list of hydroxyl and amino compounds. After removing duplicate entries, database searches were performed to annotate the chemical structures of the candidate compounds.

The annotation results were classified into five confidence levels: Level 1, the t_R_, MS1, and MS2 spectra of the candidate compound were validated using authentic standards. Level 2, the candidate compound exhibited a match with a corresponding database entry in terms of t_R_, MS1, and MS2 spectra (with an MS1 mass error < 10 ppm, a t_R_ deviation < 0.5 min, and an MS2 similarity score > 0.5). Level 3, the candidate compound matched the database entry based only on t_R_ and MS1 spectra (with an MS1 mass error < 10 ppm and a t_R_ deviation < 0.5 min). Level 4, the candidate compound matched the database entry based only on MS1 spectra (with an MS1 mass error < 10 ppm). Level 5, only the molecular formula could be determined.

### 2.9. Statistical Analysis

Partial least squares discriminant analysis (PLS-DA) was performed by SIMCA-P 13 software (Umetrics, Umea, Sweden). Statistical analyses, including *t*-tests and ANOVA, were performed using the Metware Cloud, a free online platform for data analysis (https://cloud.metware.cn, accessed on 24 December 2024). Visualization of results, including heatmaps, volcano plots, and violin plots, was also generated using the Metware Cloud.

## 3. Results

### 3.1. Characteristics of LC and MS of BrNC-Labeled Compounds

Amino and hydroxyl compounds possess nucleophilic functional groups that can be labeled through reactions with electrophilic reagents such as acyl chlorides or sulfonyl chlorides. However, under identical conditions, aliphatic hydroxyl groups—due to their relatively weak nucleophilicity—are difficult to label efficiently alongside amino and phenolic hydroxyl compounds. To date, only limited studies have reported the simultaneous derivatization of aliphatic hydroxyl, phenolic hydroxyl, and amino compounds. Zhao et al. employed dansyl chloride (DNS) to label amines, phenols, and aliphatic alcohols, but the reaction required two distinct sets of conditions [22]. Similarly, Lenhart et al. demonstrated that benzoyl chloride (BenCl) could simultaneously label amines, phenols, and certain aliphatic alcohols by optimizing reaction parameters [23]. In this study, we compared the derivatization efficiency of BrNC, DNS, and BenCl for amines and alcohols. The extracted ion chromatograms (EICs) of the derivatized products are presented in Figure S2. While all three acyl chlorides provided effective labeling for amino compounds, only BrNC successfully derivatized aliphatic alcohols. This superior reactivity may be attributed to BrNC’s enhanced electrophilicity compared to DNS and BenCl.

**Figure 1:**
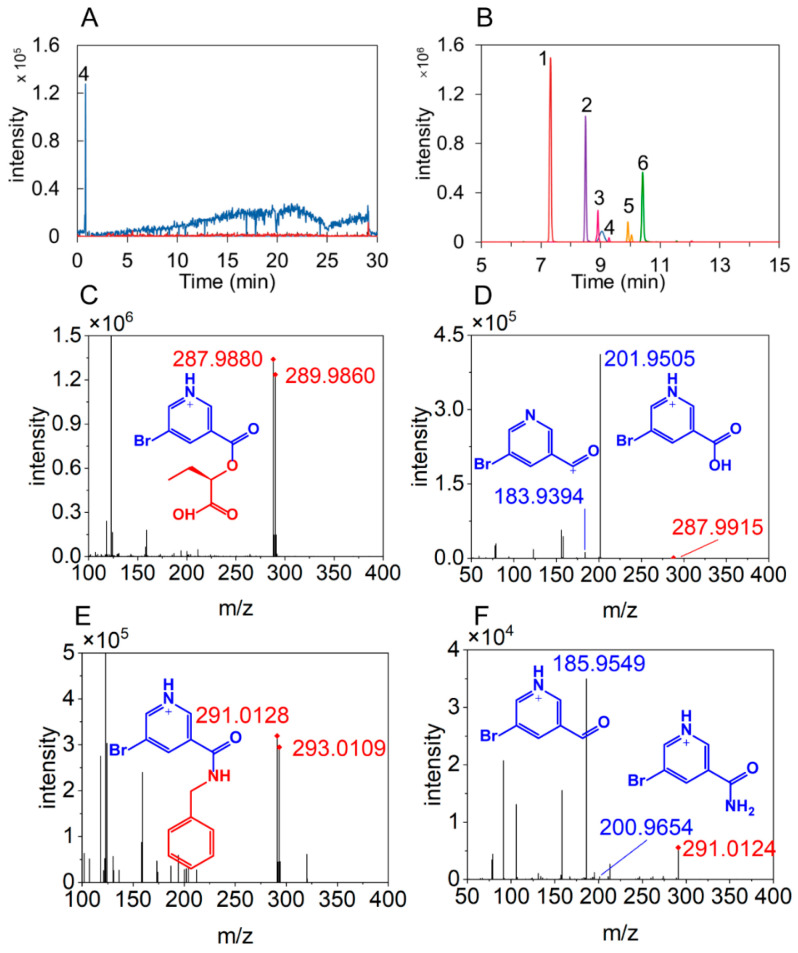
Characteristics of LC and MS of BrNC-labeled compounds. (**A**) EIC of un-labeled compounds. (**B**) EIC of labeled compounds (1. tyramine; 2. 2-hydroxybutyricacid; 3. erythritol; 4. benzenemethanamine; 5. glycerol; 6. 4-hydroxybenzaldehyde). (**C**) MS1 spectra of BrNC-labeled 2-hydroxybutyricacid. (**D**) MS2 spectrum of BrNC-labeled 2-hydroxybutyricacid. (**E**) MS1 spectra of BrNC-labeled benzylamine. (**F**) MS2 spectrum of BrNC-labeled benzylamine.

The LC and MS behavior of a series of hydroxyl and amino compounds was compared before and after BrNC-labeling. As shown in Figure 1, non-labeled hydroxyl compounds (including phenols and alcohols) are difficult to detect by direct LC-MS due to their lower ionization efficiency. However, a significant improvement in sensitivity was observed after BrNC derivatization. This enhancement can be attributed to the introduction of a tertiary amine moiety, which improves their ionization efficiency, thereby enhancing detection sensitivity. Meanwhile, although amino compounds possess strong protonation capacity, their higher polarity and hydrophilicity result in weak retention on reversed-phase liquid chromatography (RPLC) columns, leading to reduced chromatographic resolution. BrNC derivatization overcomes this limitation by incorporating a hydrophobic pyridine ring, thereby significantly improving the retention behavior of amino compounds in RPLC systems.

Furthermore, in the MS1 spectra, precursor ion pairs with a mass difference of 1.998 Da and an intensity ratio of approximately 0.97 were identified, corresponding to the natural isotopes of bromine (^78.9183^Br and ^80.9163^Br). The finding confirms that BrNC-labeled compounds can be effectively screened from a large number of precursor ions, even without the use of stable isotope-labeled reagents. Additionally, in the MS2 spectra of BrNC-labeled compounds, characteristic fragment ions were observed, including the 183.9393 fragment from ester bond cleavage, the 201.9498 fragment specific to hydroxyl compounds, and the 200.9658 fragment characteristic of amino compounds. These fragment ions facilitate the precise identification and annotation of hydroxyl and amino compounds. Collectively, BrNC derivatization facilitates highly sensitive and comprehensive detection of hydroxyl and amino compounds in complex samples.

### 3.2. Optimization of Sample Preparation Conditions

**Figure 2:**
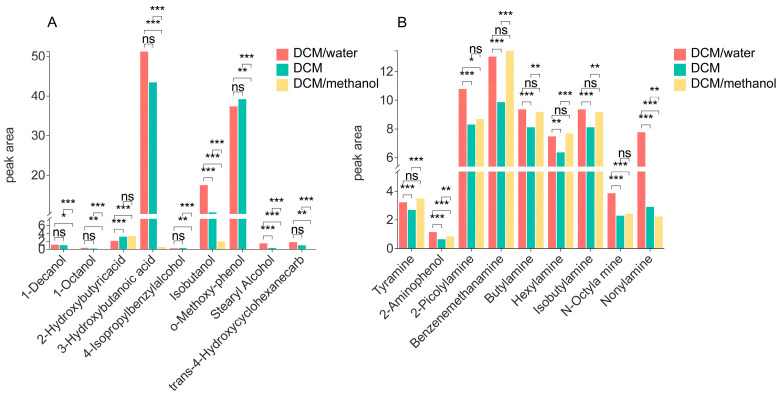
Peak area of the labeled compound determined after extraction with different solvent systems. (* *p* < 0.05, ** *p* < 0.01, *** *p* < 0.001, ns non-significant) (**A**) Hydroxyl compounds. (**B**) Amino compounds.

Given the higher ethanol and water content in Baijiu, which can significantly influence the BrNC derivatization reaction, we first evaluated the extraction methods for hydroxyl and amino compounds in Baijiu. Three parallel extractions were performed using a DCM/water mixture, DCM, and a DCM/methanol mixture, respectively. The extraction efficiency was assessed by comparing the peak area of target compounds across three extraction methods (*n* = 3 biological replicates × 2 technical injections). The statistical significance was explicitly annotated on the bar charts (Figure 2) using asterisks to indicate the level of significance (* *p* < 0.05, ** *p* < 0.01, *** *p* < 0.001). The results demonstrated that most hydroxyl compounds showed significantly higher signal responses in both DCM and DCM/water extractions compared to DCM/methanol extraction (Figure 2A). Most amino compounds exhibited significantly greater signal responses in both DCM/water and DCM/methanol extractions relative to DCM extraction (Figure 2B). Consequently, a DCM/water mixture was selected as the optimal extraction solvent to maximize the coverage of hydroxyl and amino compounds in Baijiu.

Following the optimization of the sample extraction method, the derivatization conditions for BrNC were also investigated. Given the structural similarity between BrNC and isonicotinoyl chloride (INC), which rapidly derivatizes hydroxylated steroids within 30 s under vortexing at room temperature [24], we hypothesized that BrNC would exhibit analogous derivatization reactivity. To ensure sufficient derivatization efficiency, BrNC was prepared as a 10 mg/mL suspension in anhydrous ACN, and DMAP was introduced into the reaction system as a catalyst. Subsequently, the influence of reaction time on derivatization outcomes was examined. The results demonstrated that prolonged reaction time did not improve reaction efficiency. Instead, it led to reduced derivatization yields for some compounds (Figure S3), likely attributable to BrNC hydrolysis during extended reactions. Consequently, vortexing for 30 s at room temperature was ultimately selected as the optimal BrNC derivatization condition. Notably, although sample extraction and lyophilization steps were incorporated prior to derivatization to mitigate interference from water and ethanol in Baijiu, it is recommended to use freshly prepared BrNC suspensions and anhydrous ACN for the derivatization reaction.

### 3.3. Prediction of Retention Times

To supplement chromatographic retention information in the database, a QSRR model was constructed. The QSRR_Automator software was utilized to convert the SMILES representations of compounds in the training set into a total of 371 molecular descriptors. Subsequently, a feature selection process was conducted, and 11 descriptors were identified as the most relevant for model development. Random forest regression was employed to establish the relationship between these molecular descriptors and t_R_. Following five rounds of internal cross-validation, the developed model achieved a determination coefficient (R^2^) of 0.99. Further validation of the model was conducted using an external test set. The predicted t_R_ values generated by the model were linearly fitted against the experimental t_R_ values, yielding an R^2^ of 0.98, indicating excellent agreement between the predicted and experimental values (Figure S4). The AAE was calculated to be 34 s, which further confirms the high predictive accuracy of the constructed QSRR model for the t_R_ of BrNC-derivatized compounds.

### 3.4. Method Validation

The validation results are summarized in Tables S2 and S3. The calibration curves for all 10 compounds showed good linearity (R^2^ > 0.99) across 1–4 orders of magnitude. The LOD and LOQ values ranged from 0.001–0.159 nmol/mL and 0.002–0.317 nmol/mL, respectively. The intra-day and inter-day precision for all hydroxyl and amino compounds at medium and high concentration levels demonstrated good consistency, with RSD values remaining below 10% (1.20–8.69%). At the low concentration level, all hydroxyl and amino compounds exhibited intra-day and inter-day precision with RSD values under 20% (2.80–18.02%). The results confirmed the method’s strong reliability. Furthermore, the reproducibility assessment showed that the RSDs for all tested compounds ranged from 3.20% to 18.30%, all below 20%. Regarding the stability of BrNC-labeled compounds, the peak area RSDs of all labeled compounds over 48 h were between 2.90% and 11.53%, remaining under 15%, which indicates that BrNC-labeled compounds could maintain stability when stored at 4 °C for at least two days.

Collectively, the validation results confirmed that the established BrNC-labeled method provides a stable, reproducible, and reliable result for the characterization of hydroxyl and amino compounds in Baijiu.

### 3.5. Annotation of Hydroxyl and Amino Compounds in Sauce-Flavor Baijiu

Following the annotation process described in Section 2.8, a total of 309 hydroxyl or amino compounds were identified in the mixed sample of five grades of sauce-flavor Baijiu products (SFB1, SFB2, SFB3, SFB4, and SFB5). The compounds were classified into five levels based on their annotation confidence: Level 1 (fourteen compounds), Level 2 (five compounds), Level 3 (forty-seven compounds), Level 4 (one hundred and fifty-eight compounds), and Level 5 (eighty-five compounds). The detailed information of these compounds is shown in Table S4.

**Figure 3:**
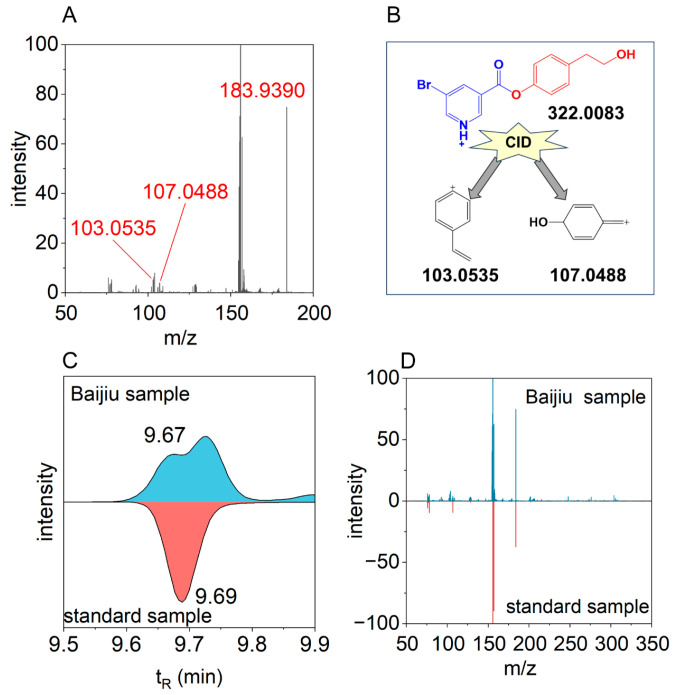
Annotation of tyrosol in Baijiu. (**A**) MS2 spectrum of precursor ion *m*/*z* 322.0083 at t_R_ =9.67 min. (**B**) Structure elucidation of fragments of *m*/*z* 322.0083. (**C**) EIC of Baijiu and standard sample. (**D**) MS2 spectra of Baijiu and standard sample.

The annotation process was exemplified using tyrosol (Figure 3). Initially, in the untargeted metabolomics data, a pair of mass spectral peaks at 9.67 min with *m*/*z* 322.0083 and *m*/*z* 324.0063 was detected, suggesting a potential BrNC-labeled compound. A database search yielded three candidate compounds that met the criteria of an MS1 deviation within 10 ppm and a t_R_ difference within 0.5 min: tyrosol, 2-hydroxymethyl-6-methylphenol, and 2,6-dimethylhydroquinone. Further analysis of the MS2 spectrum of the precursor ion revealed characteristic fragment ions of BrNC-labeled hydroxyl-containing compounds at *m*/*z* 183.9390, 201.9482, and 155.9440, along with structural fragment ions at *m*/*z* 107.0488 and 103.0535. The *m*/*z* 107.0488 fragment was consistent with methylphenol fragments, while the *m*/*z* 103.0535 fragment matched styrene-derived fragments. Based on these observations, tyrosol was considered the most probable structural assignment. Subsequent comparison with the LC-MS analysis of the tyrosol reference standard confirmed a similar t_R_ and a comparable MS2 spectrum with a similarity score of 0.71. Therefore, the compound was annotated as tyrosol with a confidence level of 1.

Collectively, the compound annotation strategy established in the study enables efficient screening of labeled compounds without requiring heavy isotope labeling. Through hierarchical annotation based on derivatization reagent fragments, MS1, MS2, and t_R_, the approach reduces false positives and provides a robust framework for investigating hydroxyl and amino metabolites in sauce-flavor Baijiu.

### 3.6. Comparison of Hydroxyl and Amino Compounds in Different Grades of Baijiu

**Figure 4:**
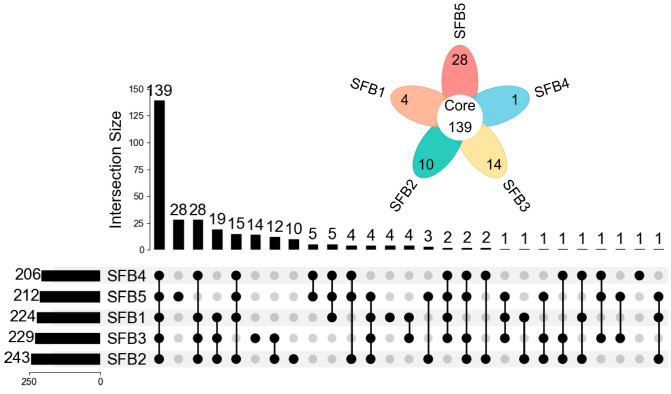
The number of hydroxyl and amino compounds detected in five grades of sauce-flavor Baijiu. The flower plot displays unique compounds (colored petals) and shared compounds (central core), while the UpSet plot details individual grade counts (left bars) and intersection patterns (top matrix).

Utilizing the established annotation strategy, 224, 243, 229, 206, and 212 hydroxyl or amino compounds were identified in SFB1, SFB2, SFB3, SFB4, and SFB5, respectively (Figure 4). Premium-grade Baijiu (SFB1-SFB3) exhibited greater compositional diversity of hydroxyl and amino compounds than lower-grade Baijiu (SFB4-SFB5), with 139 compounds in all five grades. These compounds encompass aromatic alcohols, hydroxycarboxylic acids, and phenolic derivatives (hydroxyl group), as well as amides and nitrogen-containing heterocycles (amino group).

**Figure 5:**
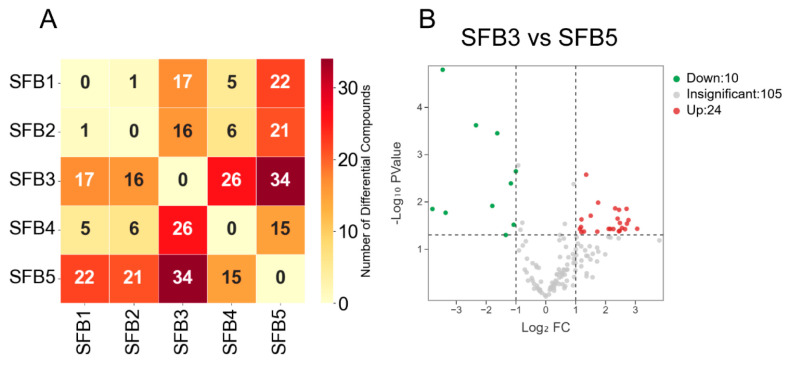
Comparison of hydroxyl and amino compounds in the five sauce-flavor Baijiu. (**A**) The number of differential compounds between each pair of the five sauce-flavor Baijiu. (**B**) Volcano plot of hydroxyl and amino compounds in SFB3 and SFB5.

Subsequent analysis of abundance variations among the 139 shared compounds revealed distinct clustering patterns via partial least squares discriminant analysis (PLS-DA), particularly between SFB3 and SFB5, indicating grade-dependent concentration differences (Figure S5). Pairwise *t*-tests further quantified inter-grade disparities (Figure 5), with SFB3 and SFB5 showing the most pronounced difference, comprising a total of 34 differential compounds. Specifically, SFB3 exhibited higher abundances of 10 compounds and lower abundances of 24 compounds than SFB5.

**Figure 6:**
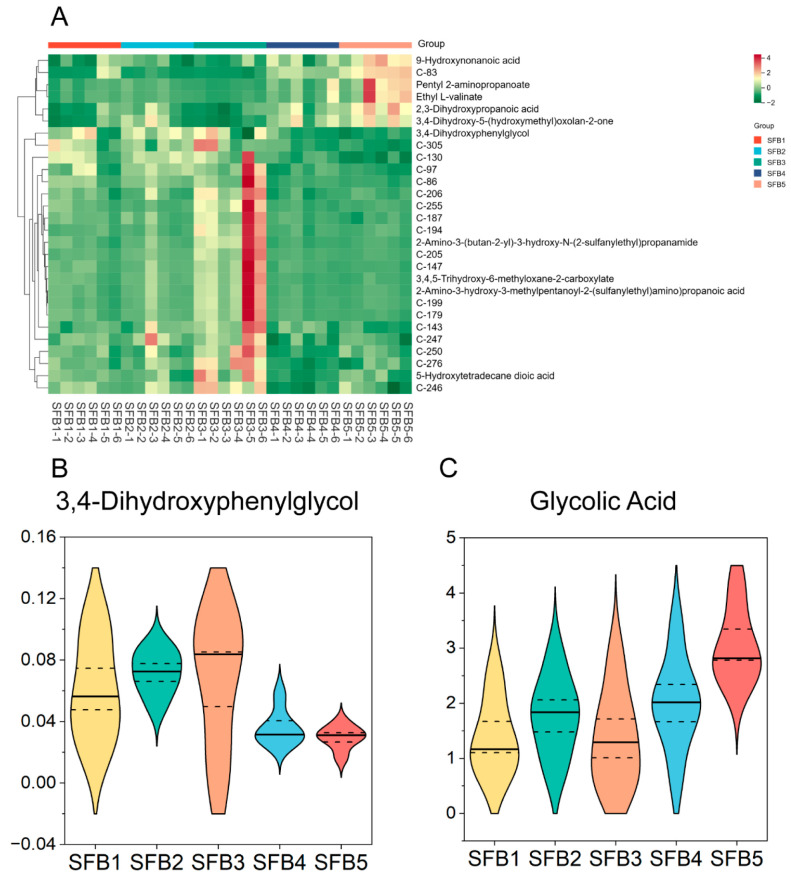
Differential compounds among the five sauce-flavor Baijiu. (**A**) Heatmap of differential compounds with VIP > 1.4. (**B**) Abundance difference of 3,4-dihydroxyphenylglycol in the five sauce-flavor Baijiu. (**C**) Abundance difference of glycolic acid in the five sauce-flavor Baijiu.

To visualize the trends, a heatmap of grade-differential compounds (variable importance in projection, VIP > 1.4) was generated (Figure 6). Several functionally relevant compounds were highlighted. For instance, 3,4-dihydroxyphenylglycol, a tyrosine-derived bioactive compound with reported anti-inflammatory and antioxidant properties [25], exhibited elevated abundances in premium grades (SFB1-SFB3). Additionally, glycolic acid, a flavor compound contributing to sourness [26], demonstrated a trend with the highest abundance in SFB5 and lowest in SFB1, potentially reflecting sensory and quality variations among different grade Baijiu.

## 4. Discussion

Sauce-flavor Baijiu contains a vast array of metabolites, among which hydroxyl and amino compounds are particularly important for defining its flavor and quality. However, detecting these compounds via LC-MS is challenging due to the poor ionization efficiency of hydroxyl compounds and the weak retention of amino compounds on reversed-phase columns. To overcome these limitations, we developed a novel BrNC derivatization strategy that significantly enhances the detection of both hydroxyl and amino compounds in Baijiu. The pyridine moiety introduced by BrNC improves compound hydrophobicity for better chromatographic separation while providing an efficient ionization site, thereby substantially boosting detection sensitivity.

Utilizing the BrNC-based derivatization approach, we comprehensively characterized 309 hydroxyl and amino compounds in sauce-flavor Baijiu. These compounds fall into four principal classes: (i) aromatic alcohols (e.g., phenethyl alcohol), (ii) hydroxycarboxylic acids (e.g., citramalic acid), (iii) phenolic derivatives, and (iv) amides and nitrogen-containing heterocycles. Notably, multiple sensorially active compounds were identified. For example, tyrosol, documented in the FooDB database (Showing Compound 2-(4-Hydroxyphenyl)ethanol (FDB012695) - FooDB), is a flavor compound characterized by floral and fruity aromas and imparts sweetness. Phenethyl alcohol, documented in the PubChem database, possesses fruity, floral, honey-like, and red wine-like notes (Phenylethyl Alcohol|C8H10O|CID 6054 - PubChem). Citramalic acid has been reported to enhance umami and sweet tastes while reducing sourness and bitterness [27]. Beyond flavor compounds, several bioactive compounds were detected in sauce-flavor Baijiu. For instance, 3-phenyllactic acid, a natural metabolite of lactic acid bacteria, has been reported to exhibit antimicrobial properties with inhibitory effects against both Gram-positive and Gram-negative bacteria [28], suggesting its potential role in flavor stabilization and quality preservation during Baijiu storage. Furthermore, 4-hydroxyphenylacetic acid was identified for the first time in sauce-flavor Baijiu. Previously reported in wine, this tyrosine-derived metabolite demonstrates gut microbiota-modulating bioactivity [29] and serves as a precursor for the flavor compound p-cresol [30].

Compared to conventional derivatization reagents such as DNS and BenCl, BrNC demonstrates superior reactivity towards both amino and hydroxyl groups (including aliphatic alcohols and phenols). This allows for efficient labeling at room temperature within 30 s without requiring extended reaction times or heating. This mild yet efficient protocol not only improves analytical throughput but also maintains the stability of hydroxyl and amino compounds during derivatization. Specifically, when compared to a previous approach using DNS derivatization requiring 1 h at 40 °C to detect 267 hydroxyl and amino compounds in Baijiu [31], our BrNC-based method achieved three key advancements: (i) 15.7% higher coverage (309 compounds identified), particularly for aliphatic alcohols that conventional reagents fail to efficiently label under mild conditions; (ii) 120 fold-faster derivatization (30 s vs. 1 h) at ambient temperature; (iii) intrinsic screening capability via bromine’s natural isotopic signature, eliminating the need for expensive isotope reagents.

The application of the established method to different grades of sauce-flavor Baijiu revealed intriguing patterns in the distribution of hydroxyl and amino compounds. Our findings indicate that both the diversity and abundance of these compounds are strongly grade-dependent, with distinct characteristics observed across different quality grades. Specifically, premium-grade Baijiu exhibited significantly greater chemical diversity and higher abundance of both flavor compounds and bioactive substances compared to lower-grade counterparts. This suggests that the compositional profiles of hydroxyl and amino compounds could serve as chemical signatures of Baijiu quality. The observed differences in compound distribution and abundance may stem from variations in raw materials and production protocols. By systematically comparing these factors among different grades, critical associations between processing conditions and compositional profiles of functional compounds could be elucidated. For instance, the higher abundance of bioactive compounds in premium-grade Baijiu may be attributed to specific fermentation conditions or higher-quality raw materials, which could enhance the stability and sensory attributes of the final product. Similarly, the elevated levels of certain flavor compounds in premium grades may reflect optimized distillation or storage processes that flavor the retention or generation of these desirable compounds.

These insights have important implications for quality control and flavor modulation in sauce-flavor Baijiu production. By identifying the specific factors that influence the diversity and abundance of hydroxyl and amino compounds, producers can make informed decisions to optimize production protocols and raw material selection. This, in turn, could lead to enhanced product consistency and improved flavor profiles, ultimately contributing to higher-quality Baijiu. Our work provides a foundation for further research into the complex interplay between production processes and the chemical composition of Baijiu, paving the way for more targeted strategies to modulate flavor and enhance quality.

## 5. Conclusions

In this study, we developed a bromine isotope labeling-based method coupled with UHPLC-HRMS for comprehensive profiling of hydroxyl and amino compounds in sauce-flavor Baijiu. A novel derivatization reagent, 5-bromonicotinoyl chloride, was designed to achieve mild and rapid labeling of hydroxyl and amino compounds. This reagent enabled efficient screening of labeled derivatives without the need for heavy isotope reagents. A total of 309 hydroxyl and amino compounds, including flavor components and bioactive substances, were identified in sauce-flavor Baijiu. The developed method demonstrated excellent utility for investigating the distribution patterns of the compounds across different quality grades of sauce-flavor Baijiu. Our findings revealed that premium-grade Baijiu exhibited a greater diversity of hydroxyl and amino compounds, a higher abundance of bioactive substances, and lower levels of acidic flavor components than lower-grade products. In summary, the developed method shows significant potential for comprehensive profiling of hydroxyl and amino compounds in sauce-flavor Baijiu. This approach could provide valuable insights for quality control and flavor modulation in Baijiu production.

## Data Availability

The data presented in this study are available in the article and Supplementary Material.

## Supplementary Materials

The following supporting information can be downloaded at https://www.mdpi.com/article/10.3390/metabo15070464/s1, Table S1: Detailed information of Baijiu samples. Table S2: Linear range, LOD, and LOQ of 10 hydroxyl and amino compounds. Table S3: Precision, repeatability, and stability of 10 hydroxyl and amino compounds. Table S4: Annotated hydroxyl and amino compounds in sauce-flavor Baijiu of Levels 1–4. Figure S1: The workflow of annotation of hydroxyl and amino compounds in Baijiu. Figure S2: EICs of compounds labeled with three derivatization reagents. Figure S3: The influence of reaction time on BrNC derivatization. Figure S4: The external validation of the established QSRR model for predicting t_R_. Figure S5. PLS-DA score plot of hydroxyl and amino compounds among the five sauce-flavor Baijiu.
