# Supplementary material for: High-Coverage Profiling of Hydroxyl and Amino Compounds in Sauce-Flavor Baijiu Using Bromine Isotope Labeling and Ultra-High Performance Liquid Chromatography–High-Resolution Mass Spectrometry
Source: Metabolites. 2025 Jul 9;15(7):464. doi: 10.3390/metabo15070464 (PMC12298331; doi:10.3390/metabo15070464)
Supplement: Supplementary file 1 [file metabolites-15-00464-s001.zip › Supporting Information.pdf]

Supporting Information

High-Coverage Profiling of Hydroxyl and Amino Compounds in Sauce-Flavor Baijiu using Bromine Isotope Labeling and Ultrahigh-Performance Liquid Chromatography-High-Resolution Mass Spectrometry

Zixuan Wang[1,3,4#], Youlan Sun[2#], Tiantian Chen[1,3,4], Lili Jiang[2], Yuhao Shang[2], Xiaolong You[2], Feng Hu[2], Di Yu[1,3,4], Xinyu Liu[1,3,4], Bo Wan[2*], Chunxiu Hu[1,3,4*], Guowang Xu[1,3,4*]

[1] State Key Laboratory of Medical Proteomics, Dalian Institute of Chemical Physics, Chinese Academy of Sciences, Dalian 116023, China

[2] Key Laboratory of Quality and Safety of Jiangxiangxing Baijiu, State Administration for Market Regulation. GuiZhou XiJiu Co., Ltd, Guizhou 564622.China.

[3] University of Chinese Academy of Sciences, Beijing 100049, China

[4] Liaoning Province Key Laboratory of Metabolomics, Dalian 116023, China

[#] Authors contributed equally to this work.

* Correspondence to:

Bo Wan, Key Laboratory of Quality and Safety of Jiangxiangxing Baijiu, State Administration for Market Regulation. GuiZhou XiJiu Co., Ltd, Guizhou 564622.China. Tel.: 15329509091, Fax: 0852-2691149. E-mail: wanboxjyj1122@outlook.com

Dr. Chunxiu Hu, State Key Laboratory of Medical Proteomics, Dalian Institute of Chemical Physics, Chinese Academy of Sciences, Dalian 116023, China. Tel.: 0086-411-84379532, Fax: 0086-411-84379559. E-mail: hucx@dicp.ac.cn

Prof. Dr. Guowang Xu, State Key Laboratory of Medical Proteomics, Dalian Institute of Chemical Physics, Chinese Academy of Sciences, Dalian 116023, China. Tel./Fax: 0086-411-84379530. E-mail: xugw@dicp.ac.cn

Table S1. Detailed information of Baijiu samples.

No.	Flavor	Manufacturer
SFB1	sauce-flavor	Guizhou Xijiu Co., LTD.
SFB2	sauce-flavor	Guizhou Xijiu Co., LTD.
SFB3	sauce-flavor	Guizhou Xijiu Co., LTD.
SFB4	sauce-flavor	Guizhou Xijiu Co., LTD.
SFB5	sauce-flavor	Guizhou Xijiu Co., LTD.

Table S2. Linear range, LOD, and LOQ of 10 hydroxyl and amino compounds.

Compounds	Linear range (ng/mL)	R^2	LOD (nmol/mL)	LOQ (nmol/mL)
2-hydroxybutyricacid	50-1250	0.998	0.087	0.174
benzyl alcohol	25-2500	0.990	0.043	0.086
6-hydroxyhexanoicacid	12.5-1250	0.999	0.016	0.040
2-hydroxyhexanoic acid	50-5000	0.991	0.159	0.317
9-hydroxynonanoic acid	1-500	0.999	0.003	0.014
nonan-1-ol	100-2500	0.991	0.038	0.306
7-hydroxyheptanoic acid	2.5-1250	0.996	0.008	0.015
4-isopropylbenzylalcohol	12.5-500	0.991	0.038	0.075
nonylamine	0.25-2500	0.991	0.001	0.003
N-octyla mine	0.5-1250	0.999	0.001	0.002

Table S3. Precision, repeatability, and stability of 10 hydroxyl and amino compounds.

Compounds	Inter-day precision (%)			Intra-day precision (%)			Repeatability (%)	Stability (48 h) (%)
	Low	Medium	High	Low	Medium	High		
2-hydroxybutyricacid	2.81	1.20	1.79	3.60	2.67	1.79	12.90	6.10
benzyl alcohol	3.25	1.97	2.66	3.25	3.09	3.63	3.82	2.90
6-hydroxyhexanoicacid	12.34	5.08	1.64	18.02	5.08	2.77	18.30	5.81
2-hydroxyhexanoic acid	10.49	4.77	8.51	10.49	4.94	8.51	12.44	7.42
9-hydroxynonanoic acid	2.90	1.72	3.21	5.58	2.64	4.44	4.72	4.22
nonan-1-ol	9.18	8.49	5.17	9.18	8.49	8.69	13.94	11.53
7-hydroxyheptanoic acid	4.78	3.20	2.27	5.97	3.20	3.84	10.49	8.77
4-isopropylbenzylalcohol	4.54	4.56	7.05	4.54	4.56	7.38	16.42	8.84
nonylamine	3.01	1.91	2.09	3.26	1.98	2.09	3.20	4.50
N-octyla mine	3.19	1.86	1.55	3.74	2.01	2.27	4.21	6.59

Table S4. Annotated hydroxyl and amino compounds in sauce-flavor Baijiu at levels 1-4 (provided in a separate Excel file).

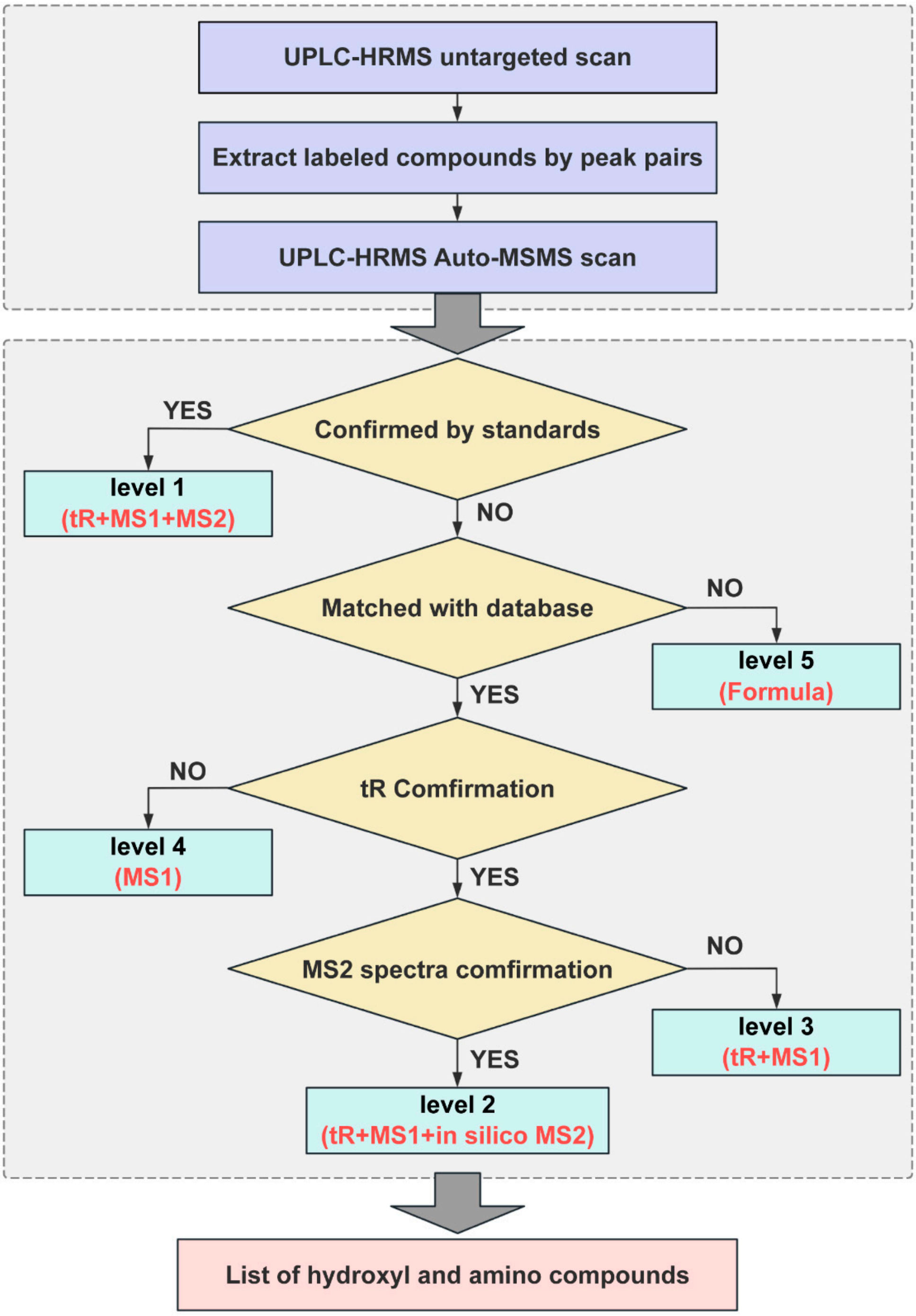

Figure S1. The annotation workflow of hydroxyl and amino compounds in Baijiu.

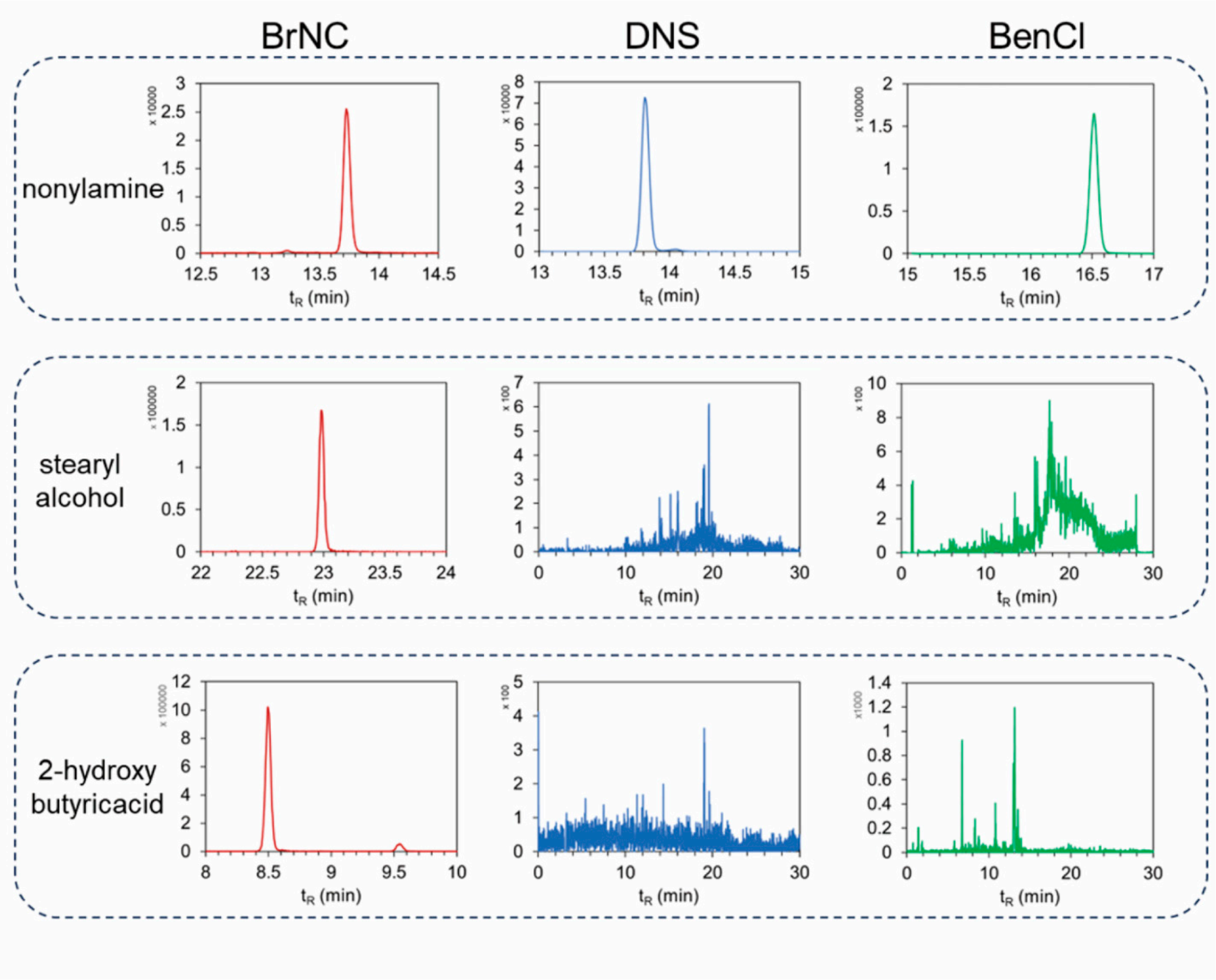

Figure S2. EICs of compounds labeled with three derivatization reagents.

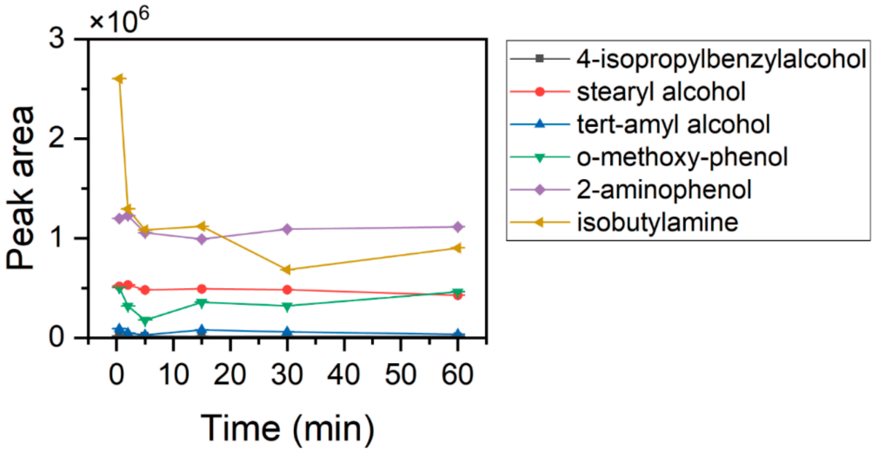

Figure S3. The influence of reaction time on BrNC derivatization.

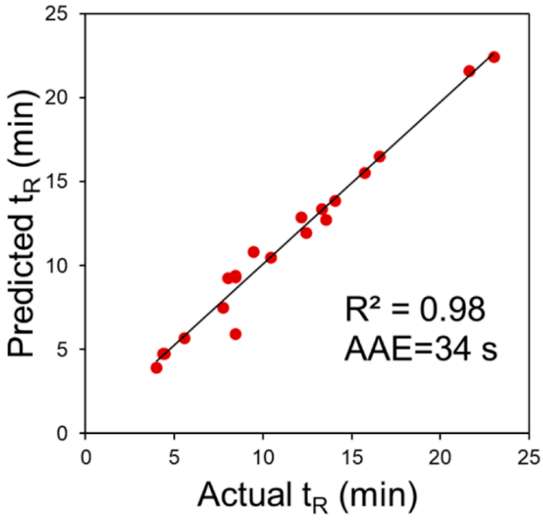

Figure S4. The external validation of the established QSRR model for predicting t_R.

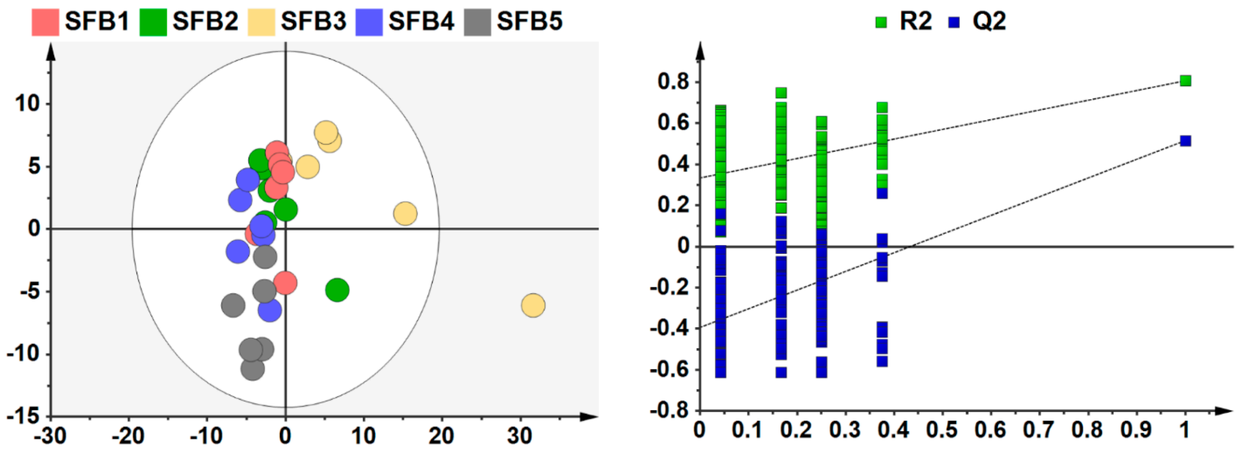

Figure S5. PLS-DA score plot of hydroxyl and amino compounds in five grades of sauce-flavor Baijiu.
